# A novel placental like alkaline phosphate promoter driven transcriptional silencing combined with single chain variable fragment antibody based virosomal delivery for neoplastic cell targeting

**DOI:** 10.1186/s12967-015-0602-1

**Published:** 2015-08-05

**Authors:** Imran Khan, Mohammad Khalid Zakaria, Mukesh Kumar, Prashant Mani, Parthaprasad Chattopadhyay, Debi P Sarkar, Subrata Sinha

**Affiliations:** Department of Biochemistry, All India Institute of Medical Sciences, New Delhi, 110029 India; National Brain Research Centre, Manesar, Gurgaon, Haryana 122051 India; Department of Biochemistry, University of Delhi, South Campus, Benito Juarez Road, New Delhi, 110021 India

**Keywords:** Placental like alkaline phosphate, Promoter, HPV-16, Transcriptional gene silencing, Recombinant antibody, scFv, Sendai virosome, Gene therapy, Germ cell alkaline phosphate (GCAP)

## Abstract

**Background:**

Placental like alkaline phosphate (PLAP), an oncofetal antigen, is highly expressed in germ cell, cervical, ovarian and several other tumour types but minimally in normal tissues. The expression of a PLAP promoter based transcriptional unit following antigen mediated cell specific delivery is a possible approach for tumour targeting.

**Methods:**

PLAP promoter alone or in combination with NFκB DNA response elements was used for expressing shRNA targeting the long control region (LCR) of human papillomavirus (HPV)-16 oncogenes E6 and E7 via transcriptional gene silencing in PLAP expressing cervical cancer cell lines, SiHa and CaSki. This was packaged in a Sendai virus envelope incorporating a single chain variable fragment antibody (scFv) for antibody mediated targeting. Specificity and efficacy of the shRNA was assessed by studying the heterochromatization, down regulation of the HPV-16 E6/E7 genes and subsequent effects on their targets and cell growth properties.

**Results:**

Reduction of HPV-16 E6 and E7 expression by TGS led to the activation of the previously suppressed target genes of p53 (PUMA and NOXA) and Rb (cyclins A2 and E). Cell death was seen only in PLAP expressing HPV-16 infected SiHa and CaSki cells but not in the HPV-18 integrated HeLa and non-PLAP CHO cells. There was reduction in the enhancer associated transcripts of the long control region (LCR) of HPV-16 E6/E7 genes. Also, an increase in the enrichment of dimethylated histone three lysine nine (H3K9Me2) and trimethylated histone three lysine twenty-seven (H3K27Me3) was observed by ChIP assay, which decreased upon trichostatin A treatment, indicating a possible mechanism for the heterochromatization of the target LCR region.

**Conclusion:**

A combination of novel PLAP promoter and antibody based specificities has the potential for being developed as a possible therapeutic strategy for PLAP positive neoplasia.

**Electronic supplementary material:**

The online version of this article (doi:10.1186/s12967-015-0602-1) contains supplementary material, which is available to authorized users.

## Background

Tumour targeting is fraught with complexities resulting from the difficulties in identifying specific attributes of neoplasia. Effective tumour management requires modalities which specifically suppress neoplastic cell programming. Tumour specific antigens like HER2/neu and CD20 have been shown to be useful for combating tumours [1–3]. In some cases such antigens have been targeted for specific delivery by antibody and particulate delivery approaches [4–6]. Neoplasia specific transcription is another avenue by which the response in tumours may be heightened. Tumour specific promoters like α-fetoprotein (AFP) to target hepatocellular carcinoma (HCC) [7], prostate-specific antigen (PSA) to target prostate cancer [8] and others [9] have been used to suppress tumour growth. We reasoned that combining an antibody based targeting modality with a construct based on antigen’s own promoter would provide a novel way for increasing tumour specificity and efficacy. Our laboratory has been working on distinct but immunologically identical oncofetal isozymes of alkaline phosphates (APs); the placental alkaline phosphate (PAP) and placental like alkaline phosphate (PLAP). In this study, we have attempted to combine a recombinant antibody based Sendai virosomal delivery system with a PLAP promoter driven shRNA for TGS.

PLAP is most frequently expressed in germ cell tumours [10], cervical cancer [11], ovarian cancer [12], but not generally in normal tissue with only a negligible degree of expression in normal cervix [13]. The ectopic expression of PLAP in malignantly transformed colon cancer cell lines has been studied by Deng and co-workers [14] who have extensively analysed PLAP promoter activity in the cells. Based on the activation of the PLAP promoter in these transformed cells, we reasoned that is could be used to provide targeted specificity to those cells where this oncofetal antigen is expressed.

A common causative factor in the etiology of cervical cancer is HPV [15]. The aggressiveness of cervical cancer is dependent on the expression of viral onco-proteins E6 and E7 [16, 17]. Transcription of both E6 and E7 in HPV-16 is governed by long control region (LCR) comprising of strong distal enhancer and weak proximal promoter p97 [18]. Therefore, targeting this region by TGS would simultaneously down regulate both E6 and E7. Conventional post-transcriptional gene silencing (PTGS) approach requires separate siRNAs against E6 and E7 transcripts; this could saturate RNAi machinery [19]. Additionally, the effects of TGS, unlike PTGS, are long lasting and genetically transmissible to the daughter cells [20]. Polymerase (pol) III promoters, due to their constitutive expression, fail to differentiate between normal and neoplastic cells; hence are not suitable for therapeutic shRNA applications [21]. To overcome this limitation, we used tumour specific PLAP promoter for expression of shRNA targeting one of the nuclear factor (NF)-1 binding site in the enhancer region of HPV-16. Further, to augment promoter mediated shRNA expression, without compromising tissue specificity, NFκB response elements and PLAP promoter were used in tandem.

Delivery of siRNA/shRNA, specifically to the cancer cell, is often limited due to lack of suitable approaches [22]. PLAP is a cancer cell membrane antigen [23] and can be manipulated for the development of tumour directed vehicular systems. Previously, we have been successful in the development of a single chain variable fragment antibody (scFv) based chimeric F-virosomal delivery system based on PAP, which is antigenically indistinguishable from PLAP [24]. In this study, TGS constructs delivered by such recombinant particulate virosomal delivery system unloaded the cargo specifically in PLAP expressing cells and produced knockdown effects only in HPV-16 infected cells SiHa and CaSki. In addition, such shRNA system induced heterochromatization of the target region without affecting the methylation pattern of CpG islands. Our results show that engineered immuno-virosomes and TGS inducing constructs provide dual specificity to tumour targeting in terms of delivery and cellular expression, hence, could be foreseen as a potential gene therapy tool.

## Methods

### Cell culture

PLAP positive cervical cancer (HeLa, SiHa and CaSki), PLAP negative hepatocellular carcinoma (HepG2) and non-PLAP, non-human Chinese hamster ovary (CHO) cell lines were used in this study. HeLa, HepG2 and CHO were obtained from American Type Culture Collection (ATCC) while SiHa and CaSki were obtained from National Centre for Cell Science (NCCS), Pune. Cells were cultured as per ATCC recommendations.

### Construction of PLAP promoter/enhancer based reporter systems

Region of the PLAP promoter previously shown to drive tissue specific expression was cloned upstream to the pGl3 Basic luciferase plasmid (Promega, USA). In order to generate a hybrid clone of the above with NFκB enhancer, ten nucleotides of NFκB enhancer sequence (10 × 4 copies) were cloned upstream to the PLAP promoter. Details of the cloning are provided in the Additional file 1. NFκBEn–Pr+24-luc and PLAPPr+24-luc generated constructs were authenticated by restriction endonuclease digestion (Additional file 1: Figure S1A) and DNA sequencing. The sequence of PLAP promoter and enhancer elements are given in Additional file 2: Figure S2A.

### Generation of TGS inducing system: PLAP promoter/enhancer + 2 HPV-16 E6/E7 shRNA

shRNA targeting NF-1 binding site on the enhancer region of HPV-16 LCR was designed using online siRNA wizard (http://www.sirnawizard.com/construct.php). 100 pico moles of sense and antisense oligonucleotides (with pre-added sticky ends; 5′*Bam*HI and 3′*Hin*dIII) were annealed as described by Zakaria et al. [25]. Details of the directional cloning strategy so that the shRNA was located downstream to the (1) PLAP promoter alone or (2) NFκB–PLAP promoter are described in Additional file 1. All the clones were confirmed by restriction digestion and authenticated by DNA sequencing before being used for transfection (Additional file 1: Figure S1B, C). This generated the constructs—NFκBEn–Pr+2-HPV-16–E6/E7, PLAPPr+2-HPV-16–E6/E7, and their appropriate scrambled controls NFκBEn–Pr+2-HPV-16–E6/E7 Scr, PLAPPr+2-HPV-16–E6/E7 Scr. shRNA under CMV promoter, CMVPr–HPV-16–E6/E7, served as positive control.

### Transfection

Cells were plated at 10^5^ cells per well in a six-well plate, 3 × 10^5^ cells per 25 cm^2^ flask or 10^6^ cells per 75 cm^2^ flask (Corning, USA). Twenty-four hours later, they were transfected with different PLAP promoter based reporter or shRNA constructs using Lipofectamine™ 2000 (Invitrogen, USA). For preparing transfectants, required amount of plasmid DNA was mixed with opti MEM media in a microfuge tube and separately Lipofectamine™ 2000 was mixed with opti MEM keeping the final volume of each tube to 50 µl. Both the tubes were incubated for about 30 min followed by transferring the contents of DNA + opti MEM to the tube containing Lipofectamine + opti MEM. The tube was incubated again for 30 min. Meanwhile, cells were washed with opti MEM media and 900 µl of opti MEM was added to each well of a 6-well plate. The contents of the tube were then added into each well. Four hours later, DMEM containing 2× serum was added and the cells were incubated. The dose of Lipofectamine™ 2000 used per/µg of plasmid was 2.5 µl. The dose of the shRNA used was 1.8 µg/well of a 6-well plate.

### Dual luciferase assay

All the three luciferase constructs: PLAPPr+24-luc, NFκBEn–Pr+24-luc and SV40-luc were transfected in a battery of cell lines. The passive lysis buffer, LAR II solution and Stop & Glo reagent were prepared as advised by the manufacturer (Promega, USA). Cells were plated onto 6-well plate and when 70% confluent, media was removed and cells were rinsed with PBS. 500 µl of passive lysis buffer was added into each well. Plate was kept on a rocker/shaker for 15 min to completely disrupt the cells. 20 µl of the resulting cell lysate was mixed with 100 µl of LAR II solution in a tube and luminescence was recorded. This was followed by addition of stop and glo solution (100 µl) and again the second readings were obtained. The firefly luciferase activity was normalized against Renilla luciferase activity and expressed relative to promoter-less pGl3-Basic control vector.

### Real-time PCR

NFκBEn–Pr+2-HPV-16–E6/E7, PLAPPr+2-HPV-16–E6/E7, CMVPr–HPV-16–E6/E7 and their appropriate scrambled controls were transfected in cell lines by following transfection protocol as described above. Trizol (Sigma-Aldrich) reagent was used for isolation of RNA at requisite time points. In order to remove DNA contamination from the extracted RNA, it was treated with DNase (MBI Fermentas) and quantified by NanoDrop ND-1000 (Thermo Fisher Scientific). About 500–1,000 ng of RNA was used for preparing cDNA by using random decamer as primers. Moloney murine leukemia virus reverse transcriptase (MBI Fermentas) was used for preparing cDNA. Real time PCR was done on a RotorGene 6000 real-time PCR machine (Corbett Research, Australia). For quantitation of target genes, we used three reference genes as an internal control—18S, GAPDH and β-actin. Relative Expression Software Tool (REST) was used for relative quantitation. The list of primers used in all experiments is given in Additional file 2.

### Cell proliferation assay

Overnight-cultured cells, 2 × 10^4^ per well, in 24-well plates, were transfected with PLAP promoter/enhancer driven shRNA constructs or their respective scrambled controls. Cell proliferation was estimated on the 6th day. On the 6th day, 10 µl of MTT reagent (Sigma Aldrich) was added to each well and the plate was incubated for 2 h. 100 µl of solubilisation buffer was added and the plate was again incubated in the dark for 2 h. 100 µl of the solution from each well was transferred onto 96-well plate and absorbance was measured at 570 nm.

### Apoptosis study

10^5^ cells were seeded in 25 cm^2^ cell culture flask (Corning, USA) followed by transfection with various shRNA constructs. On the 6th day, 70% ice-cold ethanol was utilized for fixing the cells. Propidium Iodide (PI; Sigma-Aldrich, Germany) was used for staining and Flow cytometer (BD Biosciences, USA) helped to capture the fluorescence. Cell cycle analysis was done using WinMDI software (http://winmdi.software.informer.com/2.8/)

### Western blotting

On the 6th day post transfection/immuno-virosomal delivery of shRNA constructs, cells were washed with PBS followed by lysis using triple lysis buffer [50 mmol/L Tris–Cl (pH 7.4), 150 mmol/L NaCl, 0.02% sodium azide, 0.1% SDS, 1% NP40, and 0.5% sodium deoxycholate]. Supernatants were extracted by centrifuging the lysates for 10 min. 5–12% SDS–PAGE gels were used for resolving equal quantities of protein followed by electro transfer on to nitrocellulose membranes. Blocking was done at room temperature using 4% BSA. Immunoblotting antibodies used were anti-actin (sc-8432) and anti-p53 (sc-126; Santa Cruz Biotechnology). Detection was done by ECL detection system (Applied Biosystems, USA) by using horseradish peroxidase labelled secondary antibodies.

### Preparation of chimeric scFv targeted fusion (F) Sendai virosomes and loading of shRNA constructs

This is fully described by Kumar et al. [24]. In brief, the following process went into the generation of the scFv targeted Sendai virosome. (1) The scFv antibody that had been demonstrated to bind specifically to PAP isozyme was fused in frame with a portion of the Sendai virosome’s F protein containing a segment of its membrane spanning region. (2) The virosome was then reconstituted to include both the scFv linked F protein and the wild type F protein in a ratio of 1:5. (3) The DNA constructs were incorporated as required within the virosome during the process of reconstitution of the virosome components. (4) The appropriate cells were exposed to the scFv targeted virosomes loaded with DNA constructs as described by Kumar et al. [24] and Zakaria et al. [25]. A schematic diagram is given in Additional file 3. In brief, targeting by scFv results in the juxtaposition of the virosome to the PLAP expressing cell. The membrane of the virosome and the cell then fuse as a result of the wild type F protein, which then results in direct cytoplasmic delivery of the packaged DNA constructs.

### Live cell fusion: kinetics of chimeric scFv-F-virosome fusion

1 mg/ml of Triton X-100 containing dialyzed and reduced Sendai virus envelope was mixed with 10 µl of ethanolic solution of octadecyl Rhodamine (R18) (1 mg/ml). This was vortexed and incubated in dark at room temperature for 30 min. Ultra-centrifugation, at 1,00,000*g*, was done to remove unbound R18 for 1 h at 4°C. Cells (1 × 10^6^) were incubated with 2 μg of R18 labelled scFv virosomes for 1 h at 4°C and then centrifuged at 2,000 rpm for 5 min to remove unbound virosomes. The pellet was then suspended in 100 µl of cold 10 mM PBS. 50 µl of the labeled scFv-cell complex suspension was placed in a cuvette containing 3 ml of PBS with 1.5 mM Ca^2+^ (pre-warmed to 37°C). Kinetics of fusion was recorded online by a spectrofluorimeter (Horiba, USA). This is based on dequenching of a fluorescent dye R18 after fusion, with extent of dequenching being directly proportional to the virosome cell fusion (Additional file 3).

### CpG methylation study

DNA was isolated post virosomal delivery of NFκBEn–Pr+2-HPV-16–E6/E7 or its scrambled control on the 6th day using Gen Elute Mammalian genomic DNA Miniprep Kit (Sigma-Aldrich, Germany). 500 ng of genomic DNA was bisulphite treated using EpiTect Bisulphite Kit (Qiagen, Germany). Bisulphite primers were designed from http://bisearch.enzim.hu/. Primers were M13-tagged for sequencing of PCR products.

### Chromatin immunoprecipitation (ChIP) assay

ChIP assay for H3K9Me2 and H3K27Me3 was done using EZ ChIP kit (Millipore, USA) as per manufacturer’s protocol. Immunoprecipitated DNA was amplified using primers specific for target region of HPV-16 LCR. Immunoprecipitation percentage was calculated as described earlier [26]. Cells were pre-treated with Trichostatin A (TSA; Sigma-Aldrich, Germany; 300 nM) for 48 h followed by virosomal delivery of the NFκBEn–Pr+2-HPV-16–E6/E7 or its scrambled control.

### Caspase 3/7 assay

Caspase-3/7 activity was determined post virosomal delivery of NFκBEn–Pr+2-HPV-16–E6/E7 or NFκBEn–Pr+2-HPV-16–E6/E7 Scr using caspase-3/7 assay kit (Promega, USA).

### Statistical analysis

All experiments like dual luciferase assay, cell proliferation assay and RT-PCR were performed in triplicates and repeated thrice. Western blotting, fluorescence dequenching assay, Flow cytometric analysis, Bisulfite PCR, ChIP assay and capase 3/7 assay were repeated at least twice. Student’s *t* test was utilized to calculate the significance in all experiments and p < 0.05 was considered significant whereas p < 0.001 as highly significant. The data are shown as mean ± SD.

## Results

### The transcriptional efficiency and specificity of PLAP promoter and enhancer systems

**Fig. 1:**
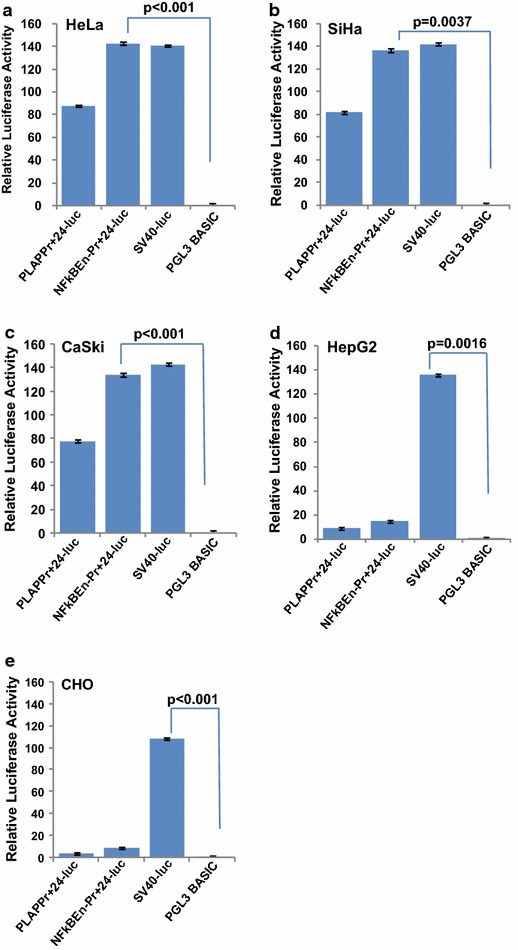
Cervical cancer specific expression of PLAP promoter/enhancer system. **a–c** 48 h after transfection, luciferase activity by enhancer/promoter system was observed only in PLAP positive cervical cancer cell lines HeLa, CaSki, and SiHa. It was highest in case of NFκBEn–Pr+24-luc. SV40-luc showed nonspecific tissue expression. **d** and **e** No, luciferase activity was observed through PLAP promoter/enhancer systems in non-PLAP HepG2 and CHO cells. Luciferase activity observed by NFκBEn–Pr+24-luc was significantly higher when compared to that by PLAPPr+24-luc (p = 0.022).

Generated luciferase constructs PLAPPr+24-luc; NFκBEn–Pr+24-luc demonstrated selective transcriptional activity only in the PLAP positive cervical cancer cell lines (HeLa, SiHa and CaSki). The transcriptional activity of NFκBEn–Pr+24-luc was comparable to that of strong SV40 promoter (SV40-luc; Fig. 1a–c; p > 0.05). However, SV40-luc also demonstrated high transcriptional activity even in PLAP negative cell lines HepG2 and CHO indicating its non-specific nature (Fig. 1d, e). Also, greater degree of luciferase expression was observed by NFκBEn–Pr+24-luc over PLAPPr+24-luc (p = 0.022).

### Reduction in E6 and E7 expression is HPV-16 specific

**Fig. 2:**
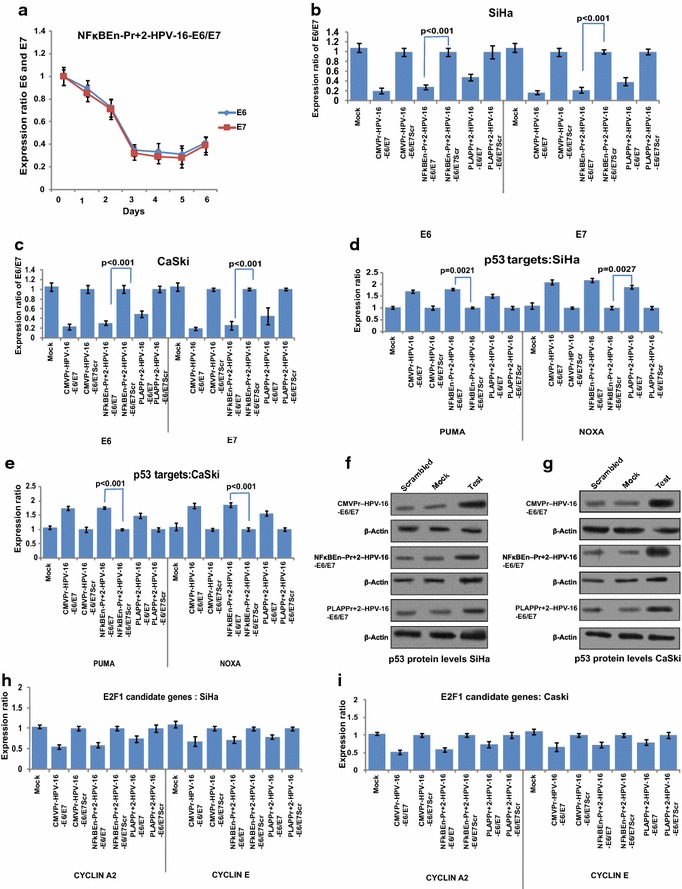
Specificity of test shRNA towards HPV-16 enhancer. **a** Time dependent fall in the expression of HPV-16 E6 and E7 by NFκBEn–Pr+2-HPV-16–E6/E7, in SiHa cells, showed maximum suppression after 5 days (p < 0.05 at all-time points). The apparent increase in E6 and E7 mRNA on the 6th day compared with 5th day was statistically insignificant (p = 0.22). **b**, **c** Decrease in E6 and E7 mRNA levels is seen in both HPV-16 positive cell lines SiHa and CaSki and the fall in E6/E7 expression is in concordance with strength of the construct driving shRNA expression. NFκBEn–Pr+2-HPV-16–E6/E7 significantly decreased HPV-16 E6/E7 mRNA levels over PLAPPr+2-HPV-16–E6/E7 in SiHa cell line (p = 0.022 and p = 0.030 for E6 and E7, respectively) and CaSki (p = 0.041 and p = 0.017 for E6 and E7, respectively). **d**, **e** Post HPV-16 E6/E7 suppression by shRNA, significant increase in the expression of p53 target genes was observed in SiHa and CaSki cells at the mRNA level**. f** and **g** Restoration of p53 protein, post HPV-16 E6/E7 suppression corroborated with the mRNA levels of PUMA and NOXA. **h** and **i** Decrease in the HPV-16 E7 expression, post shRNA treatment, significantly reduced levels of E2FI candidate genes like cyclin A2 and E (p < 0.05).

NFκBEn–Pr+2-HPV-16–E6/E7 or NFκBEn–Pr+2-HPV-16–E6/E7 Scr were transfected in SiHa cells and fall in expression of HPV-16 E6 and E7 was evaluated consecutively for 6 days This decrease was significant at all-time points (p < 0.05) and was maximum on the 5th day (Fig. 2a). Slight apparent increase on the 6th day compared to the 5th day was insignificant (p = 0.22). Fall in the HPV-16 E6 and E7 expression by other shRNA constructs in SiHa cells was also significant (Fig. 2b; p < 0.05). Similar trend was observed in CaSki cells (Fig. 2c). No significant decrease was observed in HeLa cells (p > 0.05; Additional file 4: Figure S4A) illustrating the specificity of the shRNA for HPV-16. Further, the potential to knockdown HPV-16 E6 and E7 expression by tissue specific NFκBEn–Pr+2-HPV-16–E6/E7 was comparable to tissue non-specific CMVPr–HPV-16–E6/E7 (p > 0.05). However, our NFκB–PLAP promoter, unlike CMV promoter, was active only under neoplastic condition. The activity of NFκBEn–Pr+2-HPV-16–E6/E7 was significantly higher than PLAPPr+2-HPV-16–E6/E7 in both SiHa and CaSki cells (p < 0.05). Hence, we were able to increase the transcriptional activation of the downstream TGS inducing shRNA, while retaining its tumour selective expression by fusing four copies of NFκB responsive element upstream to the PLAP promoter.

### Reduction in expression of HPV-16 E6 and E7 ameliorates p53 and abates E2FI targets

Reduction in the expression of HPV-16 E6 led to the activation of p53 as shown by increase in levels of p53 target genes like Puma and Noxa (Fig. 2d–e). This was corroborated by p53 western blot (Fig. 2f–g). The degree of HPV-16 E6/E7 suppression corroborated with the restored levels of p53 and its target genes. Hence, increased expression of p53 and its target genes was as per the strength of shRNA expression constructs: NFκBEn–Pr+2-HPV-16–E6/E7 > PLAPPr+2-HPV-16–E6/E7. Likewise, down-regulation of E7 significantly decreased the expression of E2FI candidate genes like cyclin A2 and cyclin E in SiHa and CaSki cells (Fig. 2h, i; p < 0.05).

### Suppression of HPV-16 E6 and E7 reduced cell proliferation and triggered apoptosis

**Fig. 3:**
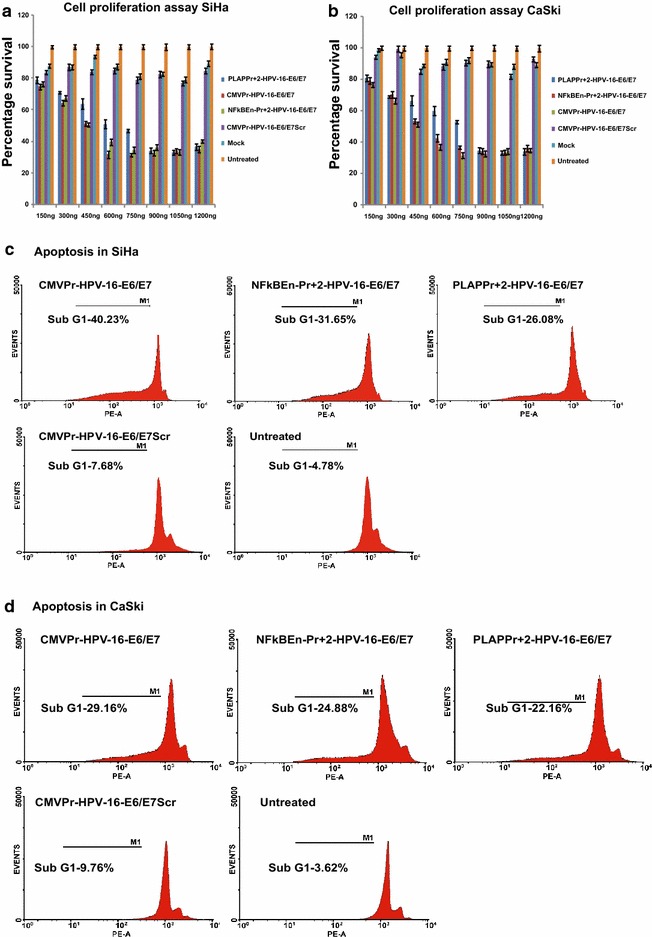
HPV-16 E6/E7 suppression reduced cell proliferation and increased apoptosis. **a** and **b** SiHa and CaSki cells were transfected with various PLAP promoter/enhancer driven test/control shRNAs in different doses and percent cell proliferation was evaluated by MTT assay on the 6th day. Decrease in cell proliferation was dependent on the dose and strength of the shRNA construct (p < 0.05). Also, NFκBEn–Pr+2-HPV-16–E6/E7, when compared to PLAPPr+2-HPV-16–E6/E7, significantly reduced cell proliferation of both SiHa and CaSki to a greater degree (p = 0.042 and p = 0.029, respectively). **c** and **d** Flow cytometry was used to evaluate apoptosis (subG1). Increase in apoptosis was concordant with the strength of promoter/enhancer construct and corroborated with the cell proliferation studies.

MTT assay revealed that there was concomitant decrease in cell proliferation of the test shRNA transfected SiHa and CaSki cells (Fig. 3a, b). Flow cytometric analysis by propidium iodide (PI) staining demonstrated increase in the percentage of cells in sub-G1 phase. This was in direct agreement with the potential of the construct expressing shRNA (Fig. 3c, d). As in the case of luciferase activity and HPV-16 E6/E7 down-regulation studies, greater degree of apoptosis and decreased cell proliferation was seen by NFκBEn–Pr+2-HPV-16–E6/E7 when compared with that by PLAPPr+2-HPV-16–E6/E7 (p < 0.05). The results observed by NFκBEn–Pr+2-HPV-16–E6/E7 were also comparable to the tissue nonspecific CMVPr–HPV-16–E6/E7 construct (p > 0.05).

### Specificity of chimeric scFv-F virosomes towards PLAP expressing cells

**Fig. 4:**
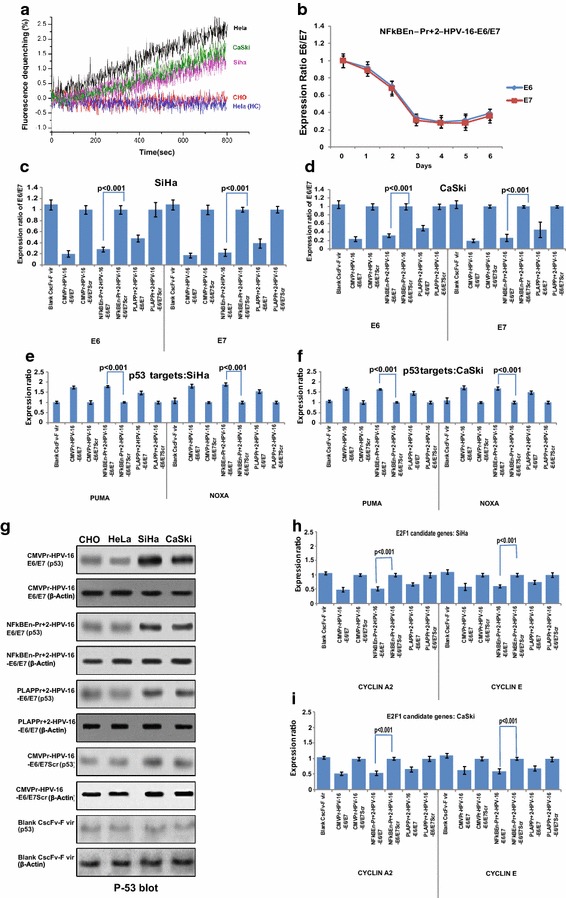
Kinetics of chimeric scFv-F-virosome fusion and knockdown effects post virosomal delivery. **a** Fusion of R18 labelled chimeric Sendai F-virosomes was determined by fluorescence dequenching assay; significant fusion was observed only in PLAP positive cells but not in non-PLAP CHO cells. scFv virosomes with inactivated F-protein (HC: heat control) displayed poor fusion with HeLa cells confirming fusion specificity via scFv. **b** Time dependant fall in expression of HPV-16 E6/E7 by NFκBEn–Pr+2-HPV-16–E6/E7, post chimeric virosomal delivery, was comparable with conventional transfection results. **c**–**f** Decrease in the expression of HPV-16 E6/E7 and increase in the expression of p53 target genes was observed in both SiHa and CaSki cells and it was in accordance with strength of the shRNA construct. **g** Amelioration in p53 in SiHa and CaSki, at the protein level, followed the same trend. **h** and **i** Post scFv F-virosomal delivery of the shRNA constructs, significant decrease in the expression of E2FI candidate genes (cyclin A2 and E) was observed in SiHa and CaSki cell lines (p < 0.05).

Real time fusion kinetics by fluorescence dequenching assay showed that the chimeric scFv-F virosomes specifically fused with PLAP positive cell lines (HeLa, CaSki and SiHa) but not with non PLAP cell line CHO which does not express PLAP. Inactivated chimeric virosomes (HC: Heat control), displayed negligible fusion with HeLa cells (Fig. 4a). The difference in the fusion observed might be dependent upon the number of PLAP molecules expressed by various cell types. Luciferase expression constructs (NFκBEn–Pr+24-luc; PLAPPr+24-luc and SV40-luc) packaged and delivered by chimeric scFv-F virosomes showed significant activity only in PLAP positive cells (Additional file 5: Figure S5A). It differed from lipofectamine based transfections as tissue non-specific SV40-luc did not elicit appreciable activity in non-PLAP cells (Fig. 1d, e). Time dependent fall in HPV-16 E6 and E7 levels, post chimeric virosomal delivery, in SiHa cells (Fig. 4b) were comparable to that by conventional methods (Fig. 2a). Significant fall in the expression of HPV-16 E6 and E7 mRNA was seen both in SiHa and CaSki cells (p < 0.05 for both; Fig. 4c, d). TGS was not effective in HeLa cells due to specificity of shRNA towards HPV-16 (Additional file 6: Figure S6A)

### HPV-16 E6 and E7 inactivation post virosomal delivery affected p53 and E2F1 candidate genes

TGS inducing constructs, following chimeric virosomal delivery, reduced the expression of HPV-16 E6 and restored the expression of p53 target genes like PUMA and NOXA (Fig. 4e, f). This was corroborated by p53 protein status (Fig. 4g). Similarly, E7 suppression was accompanied by decrease in the expression of E2FI candidate genes cyclin A2 and cyclin E in SiHa and CaSki cells but not in HeLa cells (Fig. 4h, i)

### TGS of HPV-16 E6 and E7 increased caspase 3/7 activity

**Fig. 5:**
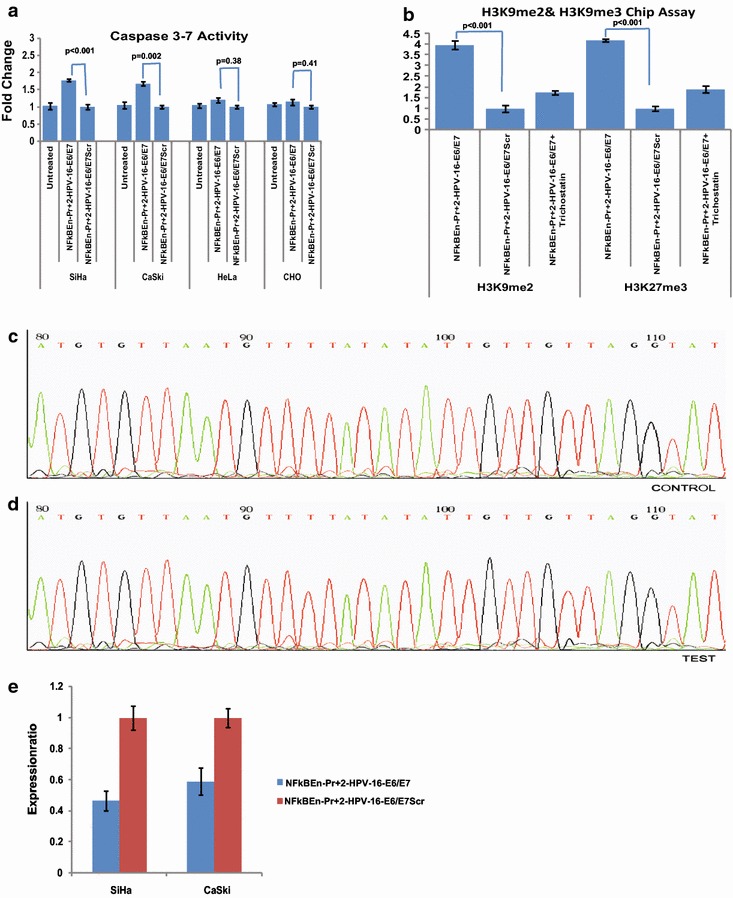
Caspase activity and mechanism involved in TGS. **a** HPV-16 E6/E7 suppression by NFκBEn–Pr+2-HPV-16–E6/E7, 5 days post virosomal delivery, led to increase in the caspase 3/7 activity in SiHa (p < 0.005) and CaSki (p = 0.02) cell lines. No such increase was observed in HeLa (p = 0.38) and CHO cells (p = 0.41). **b** Chip assay in SiHa cells transfected with NFκBEn–Pr+2 HPV-16–E6/E7 showed the silencing of the target region as a result of heterochromatization by methylation of histone tails (H3K9Me2 and H3K27Me3; p < 0.001 for both). However, cells pre-treated with TSA, did not show significant enrichment indicating that in the presence of TSA, shRNA failed to induce significant heterochromatization (p > 0.05). **c** and **d** No difference in the methylation pattern of the CpG islands, around the target LCR region, of SiHa cell line was observed by bisulphite PCR and followed by DNA sequencing. **e** The levels of enhancer associated transcripts decreased significantly post chimeric scFv-F virosomal delivery of NFκBEn–Pr+2 HPV-16–E6/E7 construct in both HPV-16 integrated SiHa and CaSki cells (p < 0.05).

Significant increase in caspase 3/7 activity was observed in SiHa and CaSki cells five days post virosomal delivery of NFκBEn+2-HPV-16–E6/E7. However, no such increase was seen in HeLa and CHO cells (Fig. 5a).

### HPV-16 shRNA induced heterochromatization without affecting CpG methylation

Using ChIP assay, the levels of repressive epigenetic marks, like H3K9me2 and H3K27me3, were found enriched, around the target region, in test shRNA treated SiHa cells. Furthermore, treatment with histone deacetylase (HDAC) inhibitor TSA reduced this enrichment indicating that HDACs are primarily involved in the process (Fig. 5b). The methylation status of CpG islands showed no change between test or control shRNA treated cells (Fig. 5c, d). This indicates that the decrease in the expression of HPV-16 E6 and E7, by shRNA treatment, is not due to DNA methylation.

### shRNA decreases the transcription of enhancer associated transcripts

The levels of the enhancer associated transcripts decreased significantly in both SiHa and CaSki after virosomal delivery of entrapped test shRNA, supporting the previously proposed RNA:RNA model of TGS [27] (Fig. 5e).

## Discussion

A tumour cell differs from a normal cell in two important characteristics—the expression of neo-antigens [28] and the transcriptional re-activation of genes that were expressed in fetal life [29]. In a way both are manifestations of the same phenomenon—the aberrant transcriptional activation of genes after neoplastic transformation results in the expression of proteins which then serve as neo-antigens. In this work we have combined both the processes for the targeting of a potentially tumour suppressive shRNA to the affected cells. The shRNA used suppresses E6/E7 genes of HPV-16 and is a potential gene therapy modality for HPV-16 mediated cervical cancer. This in itself has an element of specificity because HPV-16 is integrated only in cancer cells. However, a further degree of specificity was attempted by combining PLAP specific antigen mediated delivery with neoplasia specific PLAP promoter action. PLAP is also called germ cell alkaline phosphate (GCAP) [30]. The recombinant scFv antibody generated by us against PAP [24] also binds PLAP, which is antigenically similar and also expressed in an onco-developmental manner, but not with the other isozymes of AP. Its incorporation in the Sendai virus envelope provided specificity in delivery to the PLAP expressing transformed cells. A unique feature of this system is antibody targeted cytosolic delivery [24]. This feature enables the cargo to be delivered directly to the cytoplasm, thus bypassing endosomal uptake—a feature of the Sendai virosome envelope. Direct cytoplasmic uptake is expected to reduce degradation of the delivered cargo. The PLAP promoter, however, is distinct and the defined region [14] that provides specificity to gene expression was cloned and used either by itself or in tandem with the NFκB enhancer. As the results indicate, the objectives of specificity and effective gene expression were achieved by this strategy. The PLAP promoter has not been used so far to drive the tumour cell specific expression of potential gene therapy candidates. Hence, we attempted to suppress expression of HPV-16 oncogenes E6 and E7 via TGS induced by the PLAP promoter/enhancer driven shRNA constructs.

When measuring luciferase activity, by Dual Luciferase Assay (Fig. 1), the activity of combined NFκB enhancer and PLAP promoter (NFκBEn–Pr+24-luc) was greater than that of PLAP promoter alone (PLAPPr+24-luc; p < 0.05) in both SiHa and CaSki. However, it retained specificity for PLAP expressing cell lines. The activity of NFκBEn–Pr+24-luc was also comparable to the positive control SV40-luc (p > 0.05). But, significant SV40 mediated luciferase expression (p < 0.05) was observed even in HepG2 and CHO cells, unlike PLAP specific NFκBEn–Pr+24-luc as the SV40 promoter is ubiquitously expressed in all the cells. The above features of the naked constructs were retained after virosomal delivery.

The maintenance of the malignant aspect and behaviour of HPV transformed cervical cancer cells is dependent on the expression of viral onco-proteins E6 and E7 [16, 17]. Previously, we had used siRNA for heterochromatization of the HPV 16 LCR [31]. In this study, the specially designed PLAP promoter/enhancer driven shRNA targeting NF-1 binding site of HPV-16 LCR, reduced E6 and E7 expression only in SiHa and CaSki but not in HPV-18 integrated HeLa cell line demonstrating its specificity for HPV-16. The suppression of HPV-16 E6 and E7 oncogene expression was in concordance with the strength of each construct (Fig. 2b, c; Additional file 4: S3A). The suppression of HPV-16 E6 and E7 by tissue specific NFκBEn–Pr+2-HPV-16–E6/E7 was greater than PLAPPr+2-HPV-16–E6/E7 (p < 05; Fig. 2b, c) and was comparable to that achieved by tissue non-specific CMVPr–HPV-16–E6/E7 (p > 0.05) as the CMV promoter is tissue non-specific in nature.

The decrease in the expression of HPV-16 E6 and E7 abrogated the malignant characteristics of SiHa and CaSki but not of HPV-18 integrated HeLa cell line. This was evident by restoration in expression signatures of p53 and its target genes like Puma and Noxa and reduction in the expression profile of pRB candidate genes like cyclin A2 and cyclin E. The extent of reduction/restoration of these genes was in consonance with the strength of the promoter/enhancer driving shRNA expression (Fig. 2d, e; Additional file 4: Figure 3a, b). HPV-16 E6 and E7 suppressed cells showed decreased cell proliferation and increased apoptosis by MTT assay and flow cytometric analysis, respectively (Fig. 3a–d). In the cell proliferation and apoptosis studies, a greater decrease in the cell proliferation and increase in apoptosis was seen by NFκB–PLAP promoter driven shRNA (NFκBEn–Pr+2-HPV-16–E6/E7) when compared with that of the PLAP promoter alone (PLAPPr+2-HPV-16–E6/E7; p < 0.05).

TGS has the potential to cause long-term gene silencing by mechanisms such as heterochromatization, DNA methylation or interference with the RNA polymerase binding [25, 27, 32–35]. In our study, shRNA mediated TGS was associated with epigenetic modifications, i.e., H3K9Me2 and H3K27Me3 around the target region. The enrichment around these histones (H3K9Me2 and H3K27Me3) was reduced by TSA treatment indicating a likely involvement of HDACs (Fig. 5b). However, we observed no CpG DNA methylation suggesting that the down-regulation of HPV-16 E6/E7 occurs due to heterochromatization only (Fig. 5c). shRNA possibly acted by interacting with the enhancer associated transcripts, since significant fall in the level of these transcripts was observed post shRNA treatment (Fig. 5e). Essentially, the mechanism by which TGS was induced by current constructs was similar to what we have demonstrated earlier by siRNA [31].

The combination of antibody based targeted particulate delivery with tumour specific promoter activation has not been reported earlier. We also report for the first time the use of a region of the PLAP promoter, in tandem with the NFκB enhancer, for driving gene expression in a manner that is both specific and highly efficient in the range that can be achieved by the viral CMV promoter. This has been shown for various parameters that include the luciferase assay, ability to reduce HPV-16 E6/E7 transcript levels, reduction in cell proliferation and increase in apoptotic subG_1_ fraction. With this, we have been able to demonstrate TGS of E6 and E7 genes in HPV-16 transformed cervical carcinoma cells, leading to extensive cell death. This approach could also be utilised for the expression of beneficial genetic sequences in cancer/germ cells which ectopically or otherwise express PLAP. This combined cell delivery/transformation specific gene expression system, could serve as a paradigm for therapeutic gene delivery in malignantly transformed cells.

## Conclusions

The PLAP promoter and NFκB enhancer driven TGS inducing system, in association with the scFv directed Sendai virosome, offers a novel mode of targeting cervical cancer cells. This system could help to achieve dual cancer cell specificity firstly at the level of delivery and secondly by cancer dependent expression of the payload. This system may also be utilized in conjunction with other putative gene therapy approaches such as gene dependent enzyme prodrug therapy (GDEPT).

## Additional files

Additional file 1. Generation of various PLAP promoter/enhancer mediated constructs. Additional file 2. Sequence of the primers used in the study. Additional file 3:
**The extent of chimeric scFv-virosome fusion assessed by Fluorescence Dequencing**. The fluorophore octadecyl rhodamine beta chloride (R18) which has intrinsic quenching properties at close proximity (high concentration) was incorporated in the membrane of the virosome. When fusion with the cell occurs, the membrane fuse and mixing of target cell membrane’s and virosomal lipids takes place. This increases the distance between R18 molecules and leads to the dequenching and the fluorescence (490 excitation and 520 nm emission) is recorded with a spectrofluorimeter. Additional file 4:
**Figure S4.** Specificity of E6/E7 shRNA towards HPV-16 enhancer HPV-18 integrated cell line (HeLa) was transfected with various TGS inducing constructs (NFκBEn–Pr+2-HPV-16–E6/E7, PLAPPr+2-HPV-16–E6/E7 and CMVPr–HPV-16–E6/E7) along with their scrambled controls and the fall in expression was evaluated by real time PCR after normalization with three housekeeping genes (18s, GAPDH and β-actin). A) No, significant fall in expression of E6 and E7 mRNA was observed in HeLa, with any shRNA construct demonstrating their specificity for HPV-16 enhancer. B) There was no increase in the expression of p53 target genes like PUMA & NOXA in HeLa. C) Decrease in expression of E2FI target genes like cyclin A2 and cyclin E was not significant in HeLa. Additional file 5:
**Figure S5.** The transcriptional activity and specificity of various luciferase constructs. (A) PLAP positive HPV-18 integrated cervical cancer cell line (HeLa). (B) HPV-16 integrated cell line (SiHa). (C) HPV-18 and HPV-16 integrated cell line (CaSki). (D) PLAP negative hepatoma cell lines (HepG2). (E) Non- PLAP non-human cell line (CHO) were co-transfected by chimeric virosomes (C-scFv-V) separately in triplicates with luciferase expression vectors (PLAPPr+24–luc, SV40-luc and NFκBEn-Pr+24–luc) and Renilla expression vector (pRL-TK). The luciferase activity of each transfection was normalised by the Renilla reading. The luciferase activity is represented by the ratio of specific promoter over the activity of PGL3-Basic. No luciferase activity was observed in PLAP negative cell lines HepG2 and CHO (D and E) even by tissue nonspecific construct SV40-luc demonstrating specific delivery of packaged cargo only to PLAP expressing cells. Additional file 6:
**Figure S6.** Knockdown effects in HeLa post Immuno-virosomal delivery. Various shRNA constructs (NFκBEn–Pr+2-HPV-16–E6/E7, PLAPPr+2-HPV-16–E6/E7 and CMVPr–HPV-16–E6/E7) and their controls were packaged and delivered by chimeric virosomes to HeLa. (A) There was no significant decrease in E6 and E7 oncogene expression with any shRNA expression construct. (B) PUMA and NOXA -p53 target genes were not restored in HeLa. (C) E2F1 candidate genes were not affected post virosomal delivery of shRNA constructs in HeLa, demonstrating specificity of shRNA for HPV-16 LCR.

## Abbreviations

AFP: alpha fetoprotein
AP: alkaline phosphate
bp: base pairs
CMV: cytomegalovirus
GCAP: germ cell alkaline phosphate
HCC: hepatocellular carcinoma
HDAC: histone deacetylase
HPV: human papillomavirus
HRP: horseradish peroxidase
LCR: long control region
NF: nuclear factor
NFκB: nuclear factor-kappa B
PAP: placental alkaline phosphate
PBS: phosphate buffered saline
PAGE: polyacrylamide gel electrophoresis
PCR: polymerase chain reaction
PI: propidium iodide
PLAP: placental like alkaline phosphate
PSA: prostate specific antigen
PTGS: post transcriptional gene silencing
R18: octadecyl rhodamine beta chloride
scFv: single-chain variable fragment
SDS: sodium dodecyl sulphate
shRNA: short hairpin RNA
siRNA: small interfering RNA
SV: sendai virus
SV40: simian virus 40
TGS: transcriptional gene silencing
TSS: transcriptional start site
TSA: trichostatin A
