# Erratum to: A novel placental like alkaline phosphatase promoter driven transcriptional silencing combined with single chain variable fragment antibody based virosomal delivery for neoplastic cell targeting

**DOI:** 10.1186/s12967-015-0643-5

**Published:** 2015-09-14

**Authors:** Imran Khan, Mohammad Khalid Zakaria, Mukesh Kumar, Prashant Mani, Parthaprasad Chattopadhyay, Debi P Sarkar, Subrata Sinha

**Affiliations:** Department of Biochemistry, All India Institute of Medical Sciences, New Delhi, 110029 India; National Brain Research Centre, Manesar, Gurgaon, Haryana 122051 India; Department of Biochemistry, University of Delhi, South Campus, Benito Juarez Road, New Delhi, 110021 India

## Erratum to: J Transl Med (2015) 13:254 DOI 10.1186/s12967-015-0602-1

The original version of this article unfortunately contained a typographical mistake. In the title of the article, as well as in the body and backmatter of the article, the word ‘phosphate’ was used where ‘phosphatase’ should have been used. Please find below the instances where ‘phosphatase’ should replace ‘phosphate’ in the original article.

**Title**

A novel placental like alkaline phosphatase promoter driven transcriptional silencing combined with single chain variable fragment antibody based virosomal delivery for neoplastic cell targeting

**Abstract**

Placental like alkaline phosphatase (PLAP), an oncofetal antigen, is highly expressed in germ cell, cervical, ovarian and several other tumour types but minimally in normal tissues. The expression of a PLAP promoter based transcriptional unit following antigen mediated cell specific delivery is a possible approach for tumour targeting.

**Keywords**

Placental like alkaline phosphatase

Germ cell alkaline phosphatase (GCAP)

**Introduction**

We reasoned that combining an antibody based targeting modality with a construct based on antigen’s own promoter would provide a novel way for increasing tumour specificity and efficacy. Our laboratory has been working on distinct but immunologically identical oncofetal isozymes of alkaline phosphatases (APs); the placental alkaline phosphatase (PAP) and placental like alkaline phosphatase (PLAP).

**Discussion**

Germ cell alkaline phosphatase

**Abbreviations**

AP: alkaline phosphatase; GCAP: germ cell alkaline phosphatase; PAP: placental alkaline phosphatase; PLAP: placental like alkaline phosphatase

