# Supplementary material for: A novel placental like alkaline phosphate promoter driven transcriptional silencing combined with single chain variable fragment antibody based virosomal delivery for neoplastic cell targeting
Source: J Transl Med. 2015 Aug 5;13:254. doi: 10.1186/s12967-015-0602-1 (PMC4524171; doi:10.1186/s12967-015-0602-1)
Supplement: Additional file 1. — Generation of various PLAP promoter/enhancer mediated constructs. [file 12967_2015_602_MOESM1_ESM.pdf]

Construction of PLAP promoter/enhancer based Reporter systems

PLAP promoter+24–luciferase (PLAPPr+24–luc): Human PLAP promoter sequence [14] spanning from -170 to + 24 base pairs (bp) relative to the transcription start site (TSS) was PCR amplified from genomic DNA with primers having MluI and NheI restriction sites at 5' and 3' ends respectively. PCR amplified product was cloned into pGL3-Basic firefly luciferase reporter vector (Promega, USA) and confirmed by restriction digestion and DNA sequencing. NFκB enhancer–PLAP promoter+24–luciferase (NFκBEn-Pr+24–luc): Tetramer of NFκB response elements (5'-GGGAATTTCC-3′ x 4) were annealed and cloned upstream to the PLAP promoter in pGl3-Basic vector with 5′ KpnI and 3′ MluI sites. Clones of PLAP promoter driven luciferase reporter constructs are shown in Additional File 1: Supplementary Figure S1A. Simian virus (SV) 40 promoter driven luciferase construct (SV40–luc) served as positive control.

Generation of TGS inducing system: PLAP promoter/enhancer+2E6/E7 shRNA

PLAP and NFκB-PLAP enhancer promoter regions were amplified up to +2 bp relative to TSS from the previously generated luciferase reporter constructs (PLAPPr+24-luc; NFκBEn-Pr+24-luc respectively) with primers having5′ EcoRI and 3′ BamHI restriction sites. Amplification up to +2 bp minimizes sense strand and ensures efficient processing of shRNA. These fusion constructs were cloned along with test E6/E7 shRNA (5′ BamHI and 3′ HindIII sticky overhangs) in shRNA expression vector pSilencer 4.1 (Ambion, USA). The generated constructs were: PLAP promoter+2-E6/E7 shRNA (PLAPPr+2-HPV-16-E6/E7) and NFκB responsive element-PLAP promoter-E6/E7 shRNA (NFκBEn-Pr+2-HPV-16-E6/E7). Similarly scrambled E6/E7shRNA was cloned downstream to the promoter/enhancer constructs (PLAPPr+2-HPV-16-E6/E7 Scr; NFκBEn-Pr+2-HPV-16-E6/E7 Scr). E6/E7 test and scrambled shRNA were also

cloned under cytomegalovirus (CMV) promoter (CMVPr-HPV-16-E6/E7 and CMVPr-HPV-16-E6/E7 Scr respectively) where CMVPr-HPV-16-E6/E7 served as a positive control. Schematic representation of shRNA clones is shown in Additional File 1: Supplementary Figure S1B-C.

Supplementarary Figure 1A

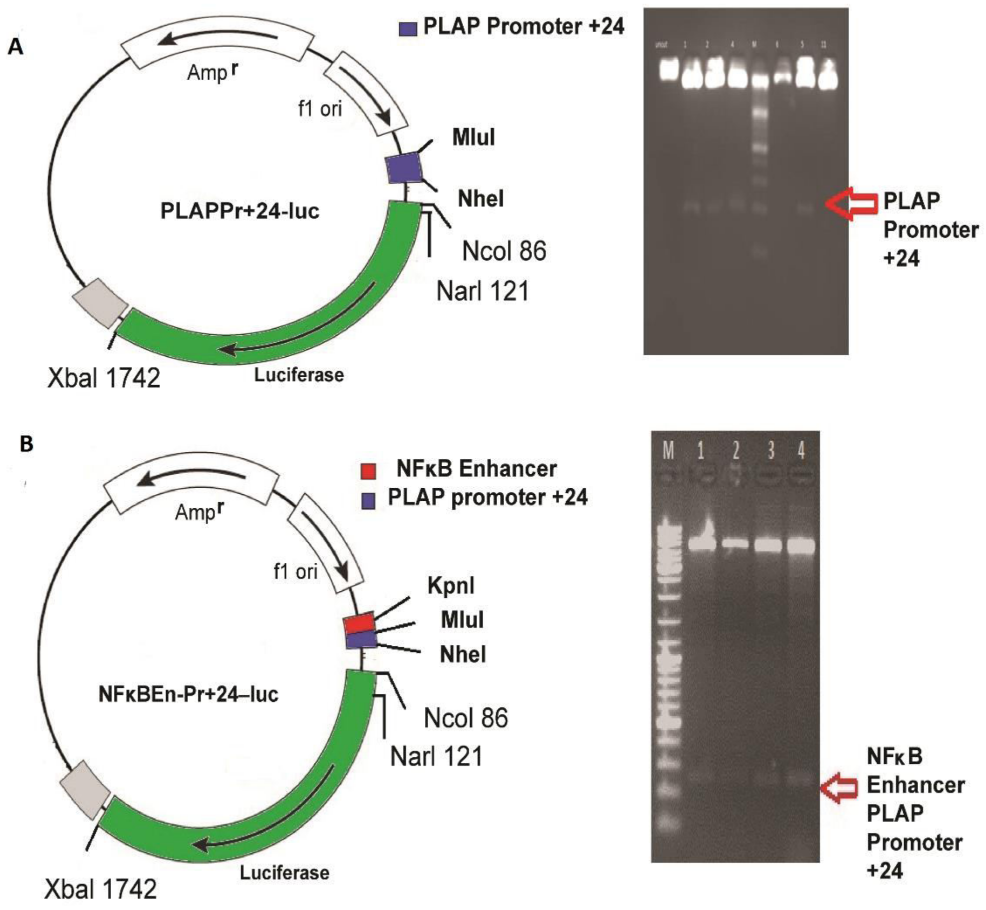

Supplementary Figure S1A: Schematic representation and confirmation of promoter and promoter enhancer based clones (**A**) PLAPPr+24 spanning from -170 to +24, cloned between *Mlu1* and *Nhe1* sites of PGL3 basic was confirmed by visualizing a band of appropriate size by double digestion with *Mlu1* and *Nhe1* (**B**) Fusion construct comprising of tetramer of 10 bp long NFκB binding site and PLAP

promoter (NFκBEn-Pr+24), cloned between *Kpn1* and *Nhe1* sites was verified by observing a band of required size by double digestion with *Kpn1 and Nhe1*.

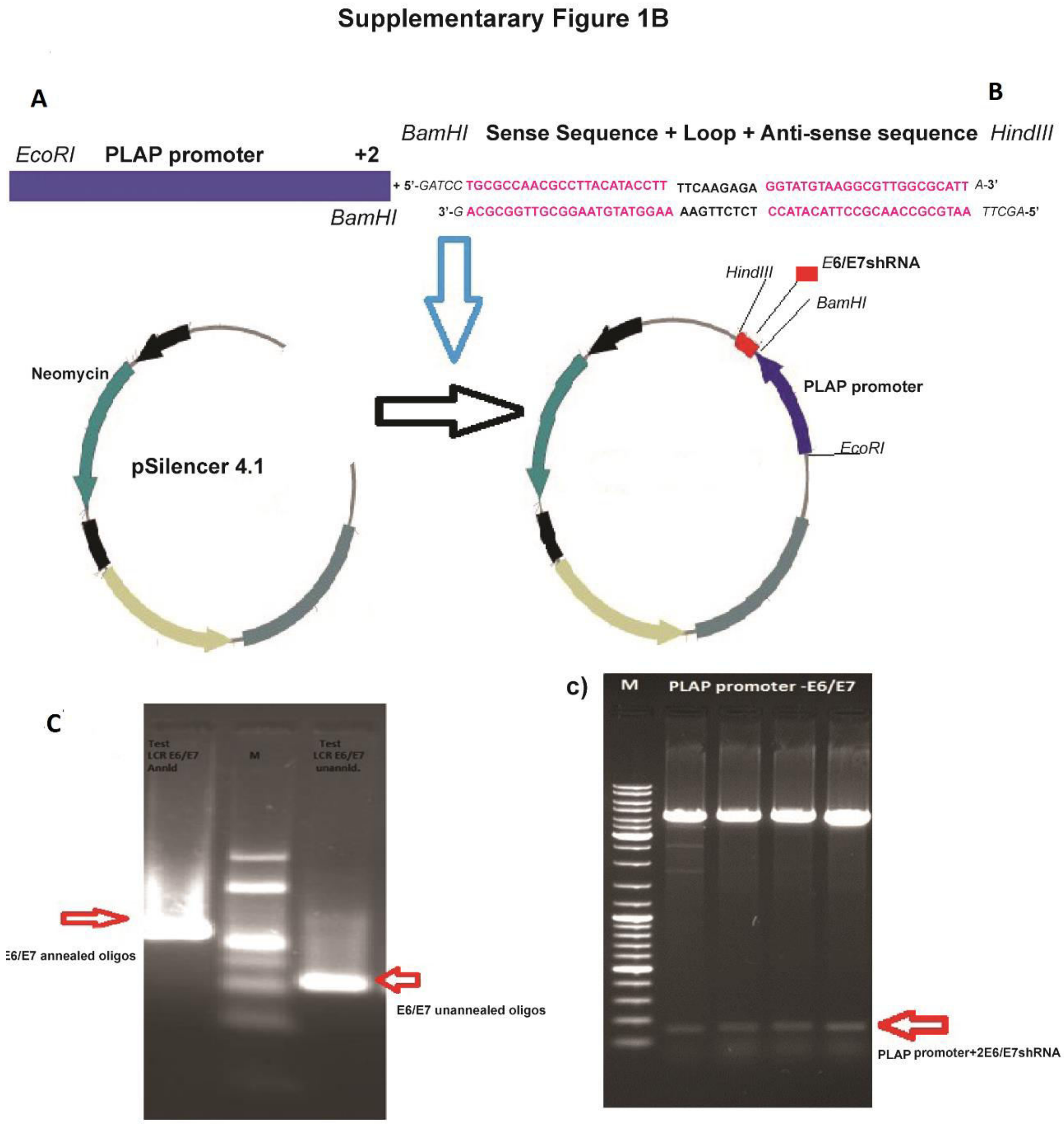

Supplementary Figure S1B: PLAP promoter–HPV-16-E6/E7 shRNA (PLAPPr+2– HPV-16-E6/E7) clone A) Schematic representation for cloning of PLAPPr+2–E6/E7 construct. **(B)** Annealed E6/E7 sense and antisense oligos. **(C)** PLAP promoter from -170 to +2 bp**,** cloned between *EcoR1* and *BamH1,* and shRNA cloned between *BamH1* and *HindII1* sites in pol (II) based shRNA vector pSilencer4.1 was confirmed by visualizing a band of appropriate size by double digestion using *EcoR1* and *HindII1* restriction endonuclease enzymes.

Supplementary Figure 1C

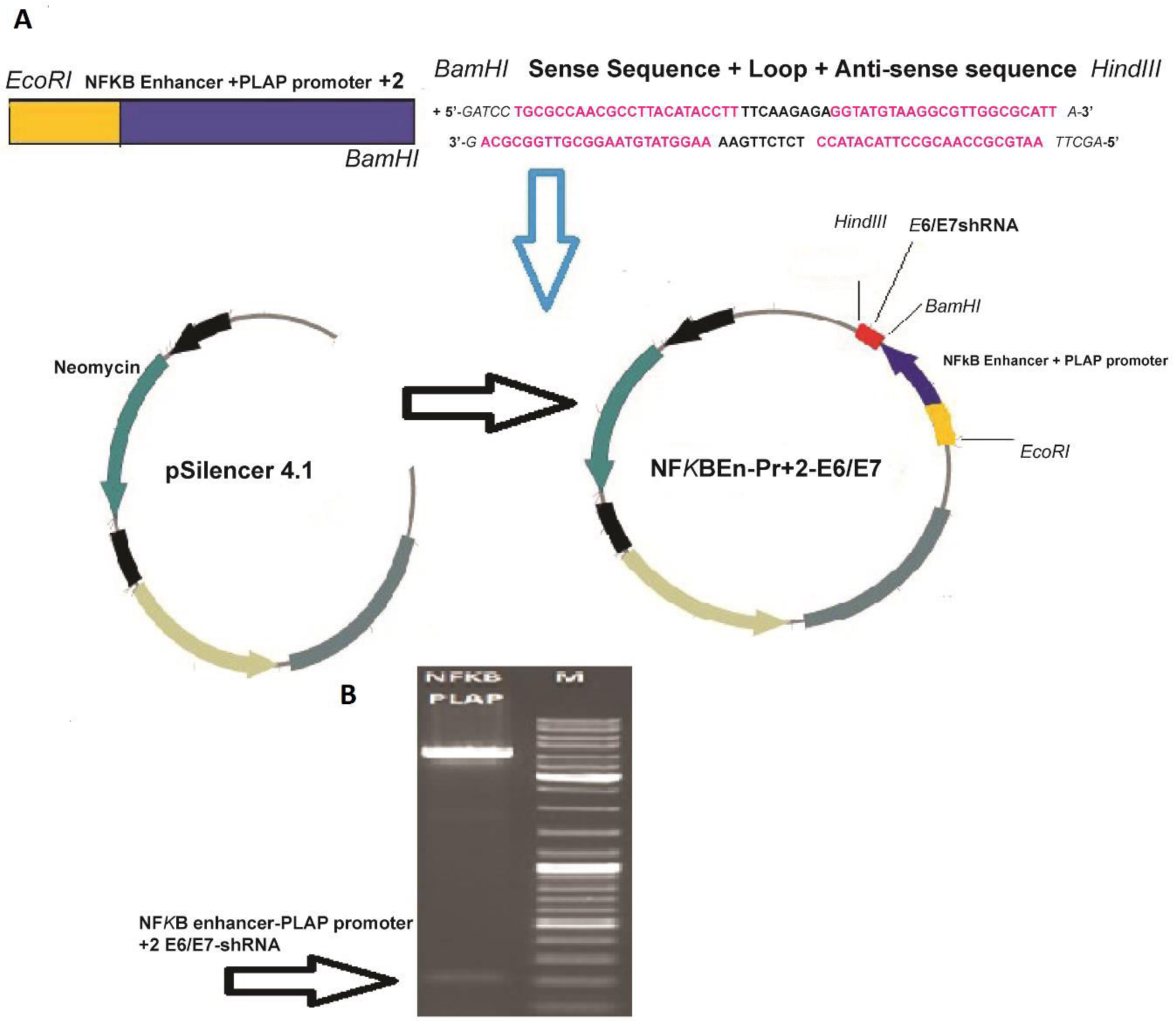

Supplementary Figure S1C: NFκB-PLAP Promoter-HPV-16-E6/E7 shRNA (NFκBEn–Pr+2–HPV-16-E6/E7) clone. A) Schematic representation for cloning of NFκBEn–Pr+2–HPV-16-E6/E7 constructs **B)** NFκB+PLAP enhancer-promoter chimera comprising of tetramer of 10 bp long NFκB binding site and PLAP promoter ranging from **-170 to +2,** cloned between *EcoR1* and *BamH1,* and shRNA cloned between *BamH1* and *HindII1* sites in pSilencer4.1 was verified by observing a band of required size by double digestion with *EcoR1* and *HindII1* restriction endonucleases.
