# Supplementary material for: A novel placental like alkaline phosphate promoter driven transcriptional silencing combined with single chain variable fragment antibody based virosomal delivery for neoplastic cell targeting
Source: J Transl Med. 2015 Aug 5;13:254. doi: 10.1186/s12967-015-0602-1 (PMC4524171; doi:10.1186/s12967-015-0602-1)
Supplement: Additional file 2. — Sequence of the primers used in the study. [file 12967_2015_602_MOESM2_ESM.pdf]

Supplementary Figure 2A

A) PLAP Promoter +24

AAGCTGCCTTTCTCAGGACCCCAGCCCCAGCCCAGCCCAGCCACACCCTGCGACT
CTCTTCAGCCAGTGTGGCTTCAGGTCAAGAGGCTGGGCGGGGTCAAGGTGGTAA
CAAGGGGAGGGGCCAGGACACAGTTTTCCCTGATTTAAACCCAGGCAGCCTGGA
GTGCAGCTCATACTCCATACCTGGGATTTCCGC

Table S1. Sequence of the primers and oligos used in the study

**Primer ID**	**Sequence(5'-3')**
**PLAP Promoter** Outer forward	AGGCCAGATGTTGGAGCA
**PLAP Promoter** Outer reverse	GCGGAAATCCCAGGTATGGAGT
**PLAP Promoter** Inner forward	CCAGCCCAGCCCAGCCACAC
**PLAP Promoter** Inner reverse	GCGGAAATCCCAGGTATGGAGT
**NFκB DNA binding sequences**	GGGAATTTCCGGGAATTTCCGGGAATTTCCGGGA ATTTCC
**E6/E7oligos**-sense strand	5'-GATCCTGCGCCAACGCCTTACATA TTCAAGAGA GGTATGTAAGGCGTTGGCGCAA-3'
**E6/E7oligos**-antisense strand	5'-AGCTTTGCGCCAACGCCTTACATACC TCTCTTGAA TATGTAAGGCGTTGGCGCA G-3'
**18S Forward**	GTAACCCGTTGAACCCCATT
**18S Reverse**	CCATCCAATCGGTAGTAGCG
**Actin Forward**	TCATGAAGTGTGACGTTGACATCCGT
**Actin Reverse**	CCTAGAAGCATTTGCGGTGCACGATG
**POLR2A** Forward	CATCAAGAGAGTCCAGTTCGG
**POLR2A** Reverse	CCCTCAGTCGTCTCTGGGTA
**PPIA** Forward	CACCGTGTTCTTCGACATTG
**PPIA** Reverse	TTCTGCTGTCTTTGGGACCT
**B2M** Forward	TAGCTGTGCTCGCGCTACT
**B2M** Reverse	TCTCTGCTGGATGACGTGAG
**Tubulin** Forward	TAACCATGAGGGAAATCGTG
**Tubulin** Reverse	TCGATGCCATGTTCATCACT

**GAPDH** Forward	CCAAGGTCATCCATGACAACTTTGGT
**GAPDH** Reverse	TGTTGAAGTCAGAGGAGACCACCTG
**HPV-16 E6** Forward	AGCGACCCAGAAAGTTACCA
**HPV-16 E6** Reverse	GCATAAATCCCGAAAAGCAA
**HPV-16 E7** Forward	ACAAGCAGAACCGGACAGAG
**HPV-16 E7** Reverse	GCCCATTAACAGGTCTTCCA
**NOXA** Forward	GAGATGCCTGGGAAGAAGG
**NOXA** Reverse	TTTCTGCCGGAAGTTCAGTT
**PUMA** Forward	GACCTCAACGCACAGTACGA
**PUMA** Reverse	CACCTAATTGGGCTCCATCT
**CyclinA2**Forward	ACCTGGACCCAGAAAACCAT
**CyclinA2**Reverse	CACTCACTGGCTTTTCATCTTC
**Cyclin E** Forward	AGA AAT GGC CAA AAT CGACA
**Cyclin E** Reverse	CCCGGTCATCATCTTCTTTG
**OAS1** Forward	TTCTCCACCTGCTTCACAGA
**OAS1** Reverse	GAGCTCCAGGGCATACTGAG
**HPV-18 E6** Forward	CATGCTGCATGCCATAAATG
**HPV-18 E6** Reverse	TGTGTTTCTCTGCGTCGTTG
**HPV-18 E7** Forward	ACCTAAGGCAACATTGCAAG
HPV-18 E7 Reverse	ACAAAGGACAGGGTGTTCAG

Primers used for Bisulphite sequencing

**Bisulphite Outer Forward**	TAGTTTTATGTTAGTAATTATGGTT
**Bisulphite Outer Reverse**	ACAACTCTATACATAACTATAATA
**Bisulphite Inner Forward** (M13 tagged)	AGCGGATAACAATTTCACACAGGATGTTTTTTTGATT TGTATTGTTTG
**Bisulphite Inner Reverse** (M13 tagged)	CGCCAGGGTTTTCCCAGTCACGACCCTTAAAAATTTA AACCTTATACC

Primers used for ChIP

**Chr16ChIP Forward**	GTCTCTTTCTTGTTTTTAAGCTGGG
**Chr16ChIP Reverse**	TGAGCTCATTGAGACATTTGG
**HPV-16 LCR Forward**	CCAAATCCCTGTTTTCCTGA
**HPV-16 LCR Reverse**	CGTTGGCGCATAGTGATTTA

Supplementary Figure S2A: (A) Sequence of the PLAP promoter used in the study. **(B)** List of Primers used.
