# Supplementary material for: A novel placental like alkaline phosphate promoter driven transcriptional silencing combined with single chain variable fragment antibody based virosomal delivery for neoplastic cell targeting
Source: J Transl Med. 2015 Aug 5;13:254. doi: 10.1186/s12967-015-0602-1 (PMC4524171; doi:10.1186/s12967-015-0602-1)
Supplement: Additional file 3: — The extent of chimeric scFv-virosome fusion assessed by Fluorescence Dequencing. The fluorophore octadecyl rhodamine beta chloride (R18) which has intrinsic quenching properties at close proximity (high concentration) was incorporated in the membrane of the virosome. When fusion with the cell occurs, the membrane fuse and mixing of target cell membrane’s and virosomal lipids takes place. This increases the distance between R18 molecules and leads to the dequenching and the fluorescence (490 excitation and 520 nm emission) is recorded with a spectrofluorimeter. [file 12967_2015_602_MOESM6_ESM.pdf]

A)

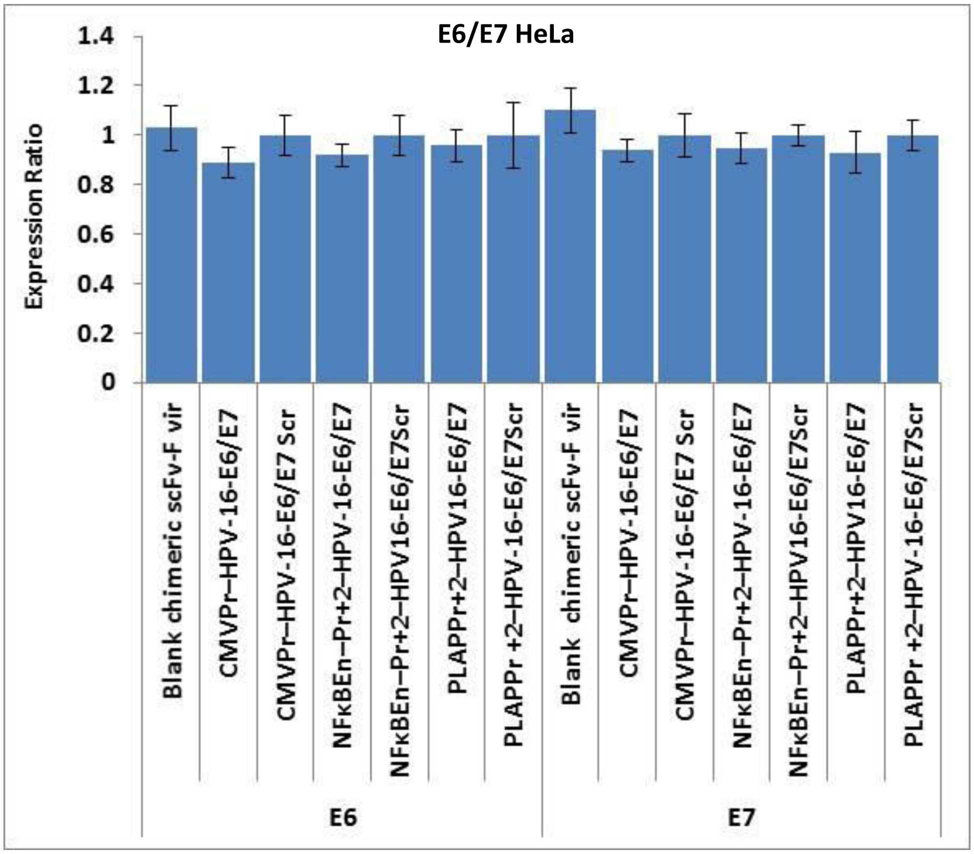
E6/E7 HeLa
Expression Ratio
1.4
1.2
1
0.8
0.6
0.4
0.2
0
Blank chimeric scFv-F vir
CMVPr–HPV-16-E6/E7
CMVPr–HPV-16-E6/E7 Scr
NFκBEn–Pr+2–HPV-16-E6/E7
NFκBEn–Pr+2–HPV16-E6/E7Scr
PLAPPr+2–HPV16-E6/E7
PLAPPr +2–HPV-16-E6/E7Scr
Blank chimeric scFv-F vir
CMVPr–HPV-16-E6/E7
CMVPr–HPV-16-E6/E7 Scr
NFκBEn–Pr+2–HPV-16-E6/E7
NFκBEn–Pr+2–HPV16-E6/E7Scr
PLAPPr+2–HPV16-E6/E7
PLAPPr +2–HPV-16-E6/E7Scr
E6
E7

B)

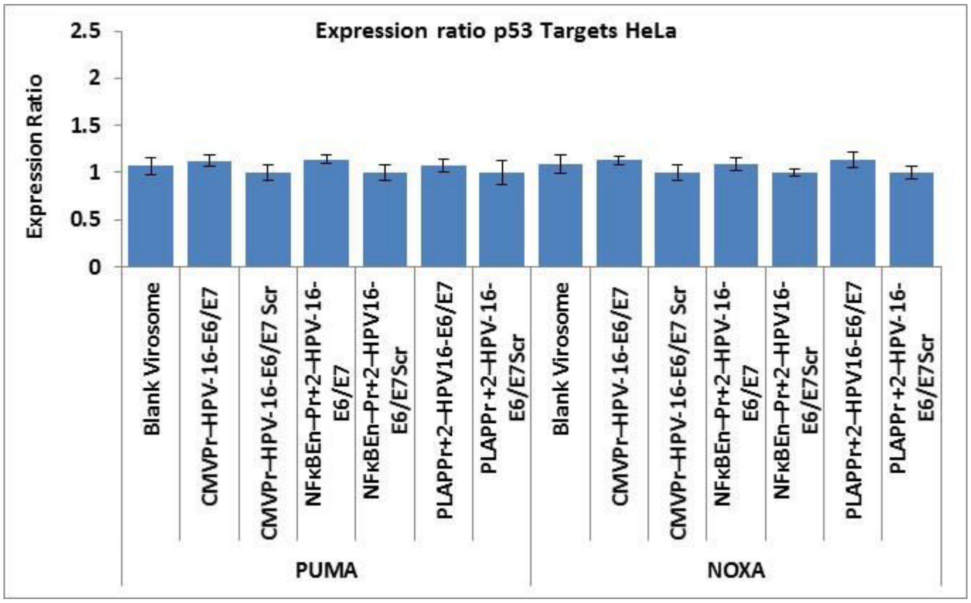
Expression ratio p53 Targets HeLa
Expression Ratio
2.5
2
1.5
1
0.5
0
Blank Virosome
CMVPr–HPV-16-E6/E7
CMVPr–HPV-16-E6/E7 Scr
NFκBEn–Pr+2–HPV-16-E6/E7
NFκBEn–Pr+2–HPV16-E6/E7Scr
PLAPPr+2–HPV16-E6/E7
PLAPPr +2–HPV-16-E6/E7Scr
Blank Virosome
CMVPr–HPV-16-E6/E7
CMVPr–HPV-16-E6/E7 Scr
NFκBEn–Pr+2–HPV-16-E6/E7
NFκBEn–Pr+2–HPV16-E6/E7Scr
PLAPPr+2–HPV16-E6/E7
PLAPPr +2–HPV-16-E6/E7Scr
PUMA
NOXA

**C)**

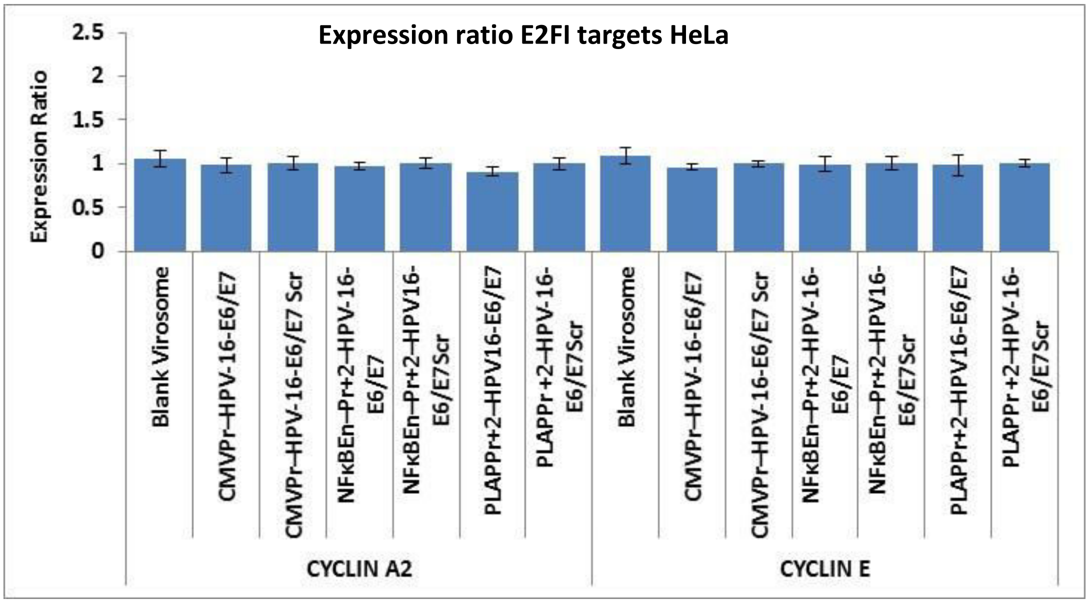


**Supplementary Figure S6A: Knockdown effects in HeLa post Immuno-virosomal delivery.** Various shRNA constructs (NFκBEn–Pr+2–HPV-16-E6/E7, PLAPPr+2-HPV-16–E6/E7 and CMVPr-HPV-16-E6/E7) and their controls were packaged and delivered by chimeric virosomes to HeLa. **(A)** There was no significant decrease in E6 and E7 oncogene expression with any shRNA expression construct. **(B)** PUMA and NOXA -p53 target genes were not restored in HeLa. **(C)** E2F1 candidate genes were not affected post virosomal delivery of shRNA constructs in HeLa, demonstrating specificity of shRNA for HPV-16 LCR
