# Supplementary material for: A novel placental like alkaline phosphate promoter driven transcriptional silencing combined with single chain variable fragment antibody based virosomal delivery for neoplastic cell targeting
Source: J Transl Med. 2015 Aug 5;13:254. doi: 10.1186/s12967-015-0602-1 (PMC4524171; doi:10.1186/s12967-015-0602-1)
Supplement: Additional file 4: — Figure S4. Specificity of E6/E7 shRNA towards HPV-16 enhancer HPV-18 integrated cell line (HeLa) was transfected with various TGS inducing constructs (NFκBEn–Pr+2-HPV-16–E6/E7, PLAPPr+2-HPV-16–E6/E7 and CMVPr–HPV-16–E6/E7) along with their scrambled controls and the fall in expression was evaluated by real time PCR after normalization with three housekeeping genes (18s, GAPDH and β-actin). A) No, significant fall in expression of E6 and E7 mRNA was observed in HeLa, with any shRNA construct demonstrating their specificity for HPV-16 enhancer. B) There was no increase in the expression of p53 target genes like PUMA & NOXA in HeLa. C) Decrease in expression of E2FI target genes like cyclin A2 and cyclin E was not significant in HeLa. [file 12967_2015_602_MOESM3_ESM.pdf]

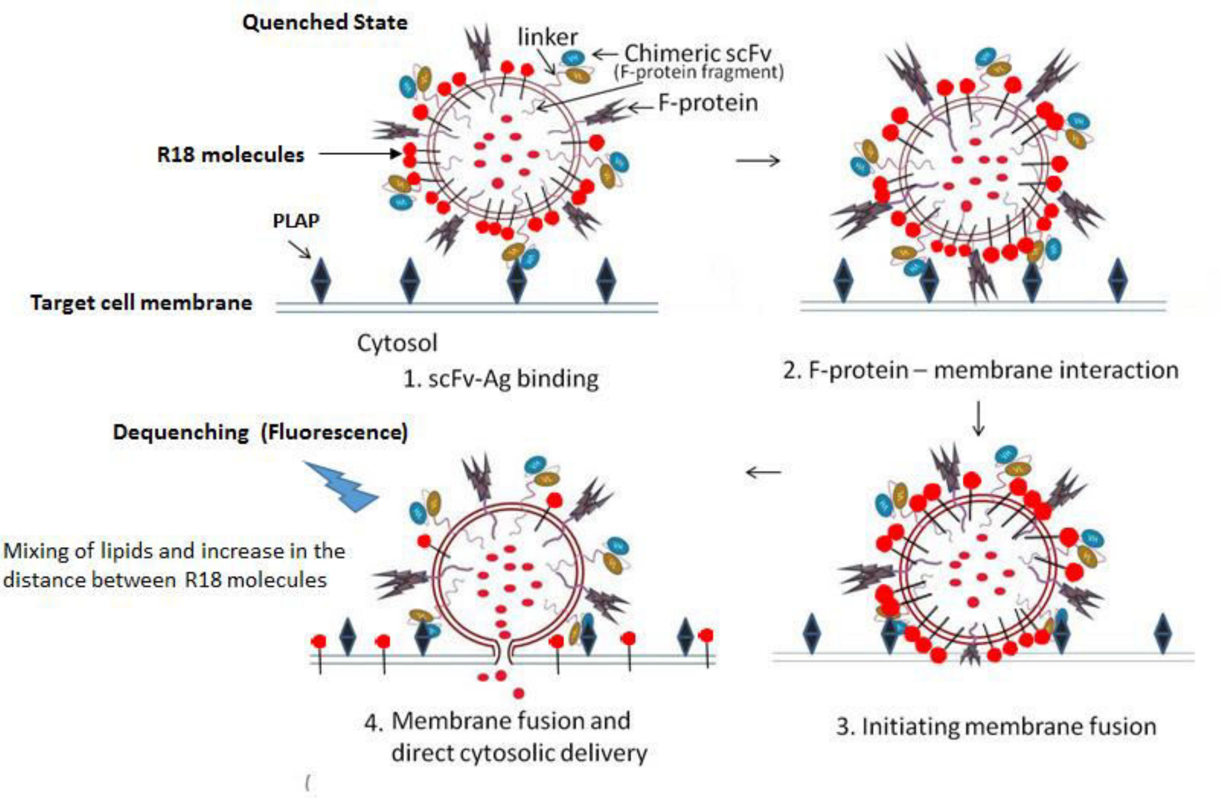

Additional File 3: The extent of chimeric scFv-virosome fusion assessed by Fluorescence Dequencing. The fluorophore octadecyl rhodamine beta chloride (R18) which has intrinsic quenching properties at close proximity (high concentration) was incorporated in the membrane of the virosome. When fusion with the cell occurs, the membrane fuse and mixing of target cell membrane's and virosomal lipids takes place. This increases the distance between R18 molecules and leads to the dequenching and the fluorescence (490 excitation and 520 nm emission) is recorded with a spectrofluorimeter.
