# Supplementary material for: A novel placental like alkaline phosphate promoter driven transcriptional silencing combined with single chain variable fragment antibody based virosomal delivery for neoplastic cell targeting
Source: J Transl Med. 2015 Aug 5;13:254. doi: 10.1186/s12967-015-0602-1 (PMC4524171; doi:10.1186/s12967-015-0602-1)
Supplement: Additional file 5: — Figure S5. The transcriptional activity and specificity of various luciferase constructs. (A) PLAP positive HPV-18 integrated cervical cancer cell line (HeLa). (B) HPV-16 integrated cell line (SiHa). (C) HPV-18 and HPV-16 integrated cell line (CaSki). (D) PLAP negative hepatoma cell lines (HepG2). (E) Non- PLAP non-human cell line (CHO) were co-transfected by chimeric virosomes (C-scFv-V) separately in triplicates with luciferase expression vectors (PLAPPr+24–luc, SV40-luc and NFκBEn-Pr+24–luc) and Renilla expression vector (pRL-TK). The luciferase activity of each transfection was normalised by the Renilla reading. The luciferase activity is represented by the ratio of specific promoter over the activity of PGL3-Basic. No luciferase activity was observed in PLAP negative cell lines HepG2 and CHO (D and E) even by tissue nonspecific construct SV40-luc demonstrating specific delivery of packaged cargo only to PLAP expressing cells. [file 12967_2015_602_MOESM4_ESM.pdf]

A)

Hela E6/E7

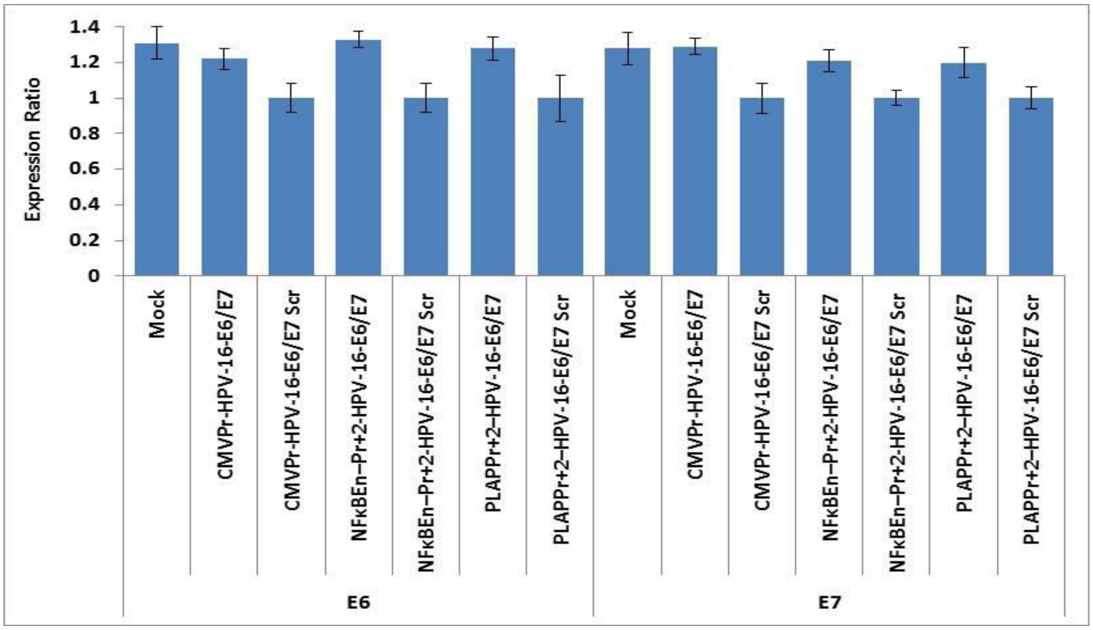
Expression Ratio
1.4
1.2
1
0.8
0.6
0.4
0.2
0
Mock
CMVPr-HPV-16-E6/E7
CMVPr-HPV-16-E6/E7 Scr
NFκBEn–Pr+2-HPV-16-E6/E7
NFκBEn–Pr+2-HPV-16-E6/E7 Scr
PLAPPr+2–HPV-16-E6/E7
PLAPPr+2–HPV-16-E6/E7 Scr
E6
E7

B)

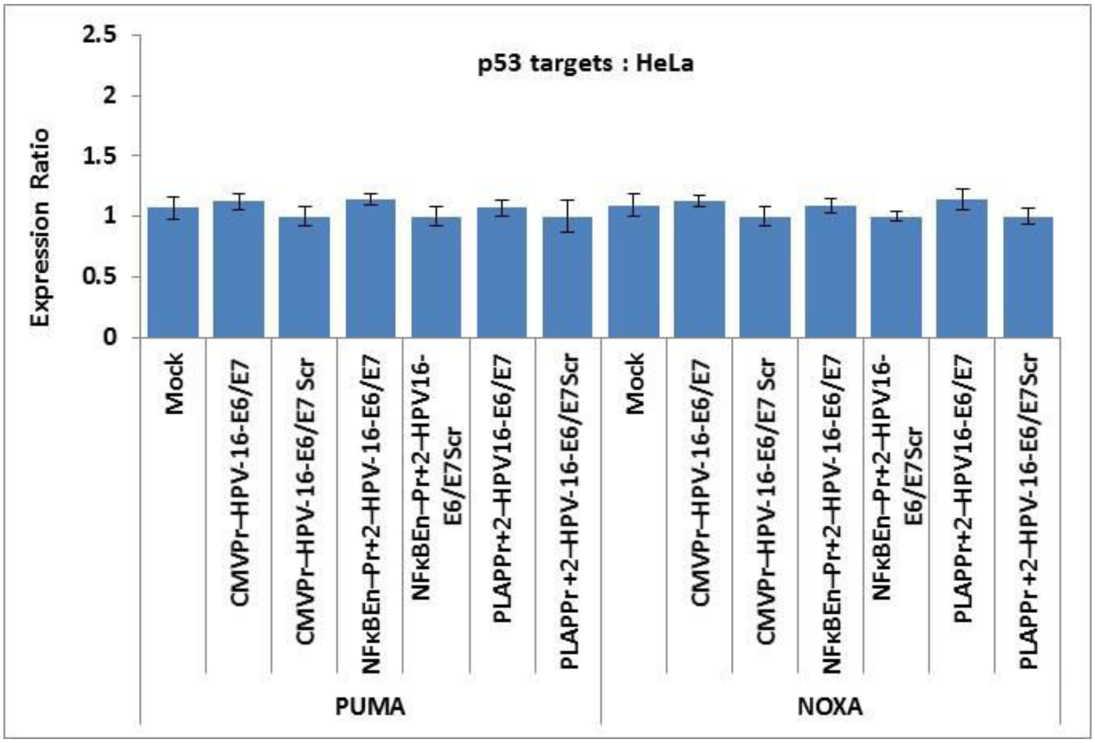
p53 targets : HeLa
Expression Ratio
2.5
2
1.5
1
0.5
0
Mock
CMVPr–HPV-16-E6/E7
CMVPr–HPV-16-E6/E7 Scr
NFκBEn–Pr+2–HPV-16-E6/E7
NFκBEn–Pr+2–HPV16-E6/E7Scr
PLAPPr+2–HPV16-E6/E7
PLAPPr+2–HPV-16-E6/E7Scr
PUMA
NOXA

C)

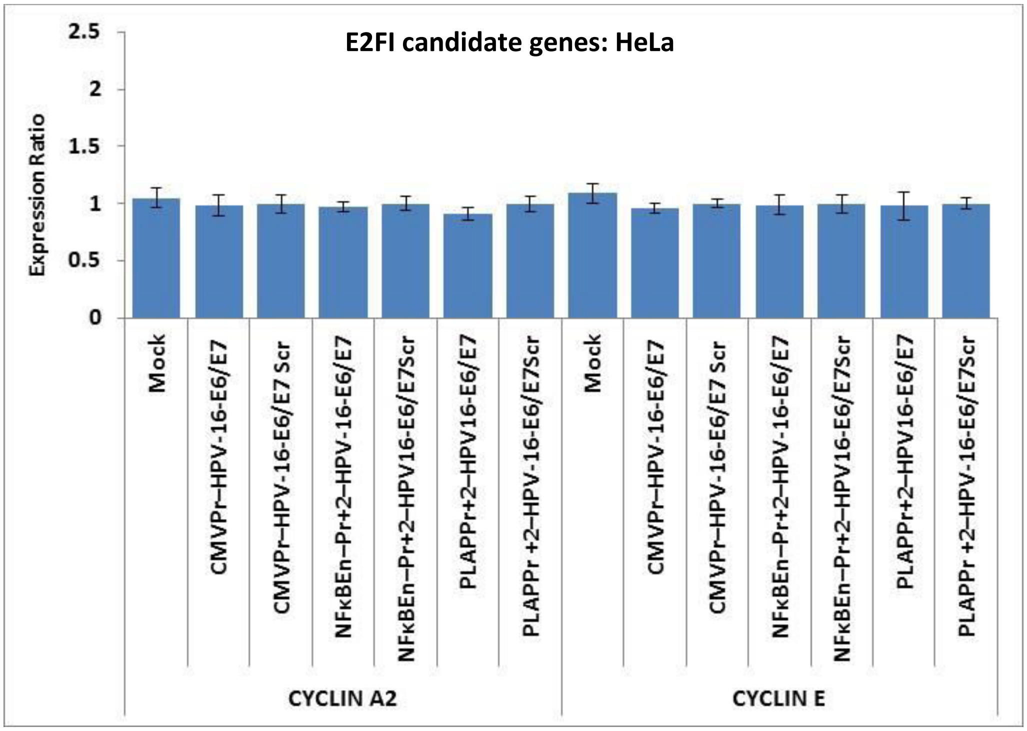

Supplementary Figure S4A: Specificity of E6/E7 shRNA towards HPV-16 enhancer HPV-18 integrated cell line (HeLa) was transfected with various TGS inducing constructs (NFκBEn-Pr+2-HPV-16-E6/E7, PLAPPr+2-HPV-16-E6/E7 and CMVPr-HPV-16-E6/E7) along with their scrambled controls and the fall in expression was evaluated by real time PCR after normalization with three housekeeping genes(18s,GAPDH and β-actin). **A)** No, significant fall in expression of E6 and E7 mRNA was observed in HeLa, with any shRNA construct demonstrating their specificity for HPV-16 enhancer. **B)** There was no increase in the expression of p53 target genes like PUMA & NOXA in HeLa. **C)** Decrease in expression of E2FI target genes like cyclin A2 and cyclin E was not significant in HeLa.
