# Supplementary material for: A novel placental like alkaline phosphate promoter driven transcriptional silencing combined with single chain variable fragment antibody based virosomal delivery for neoplastic cell targeting
Source: J Transl Med. 2015 Aug 5;13:254. doi: 10.1186/s12967-015-0602-1 (PMC4524171; doi:10.1186/s12967-015-0602-1)
Supplement: Additional file 6: — Figure S6. Knockdown effects in HeLa post Immuno-virosomal delivery. Various shRNA constructs (NFκBEn–Pr+2-HPV-16–E6/E7, PLAPPr+2-HPV-16–E6/E7 and CMVPr–HPV-16–E6/E7) and their controls were packaged and delivered by chimeric virosomes to HeLa. (A) There was no significant decrease in E6 and E7 oncogene expression with any shRNA expression construct. (B) PUMA and NOXA -p53 target genes were not restored in HeLa. (C) E2F1 candidate genes were not affected post virosomal delivery of shRNA constructs in HeLa, demonstrating specificity of shRNA for HPV-16 LCR. [file 12967_2015_602_MOESM5_ESM.pdf]

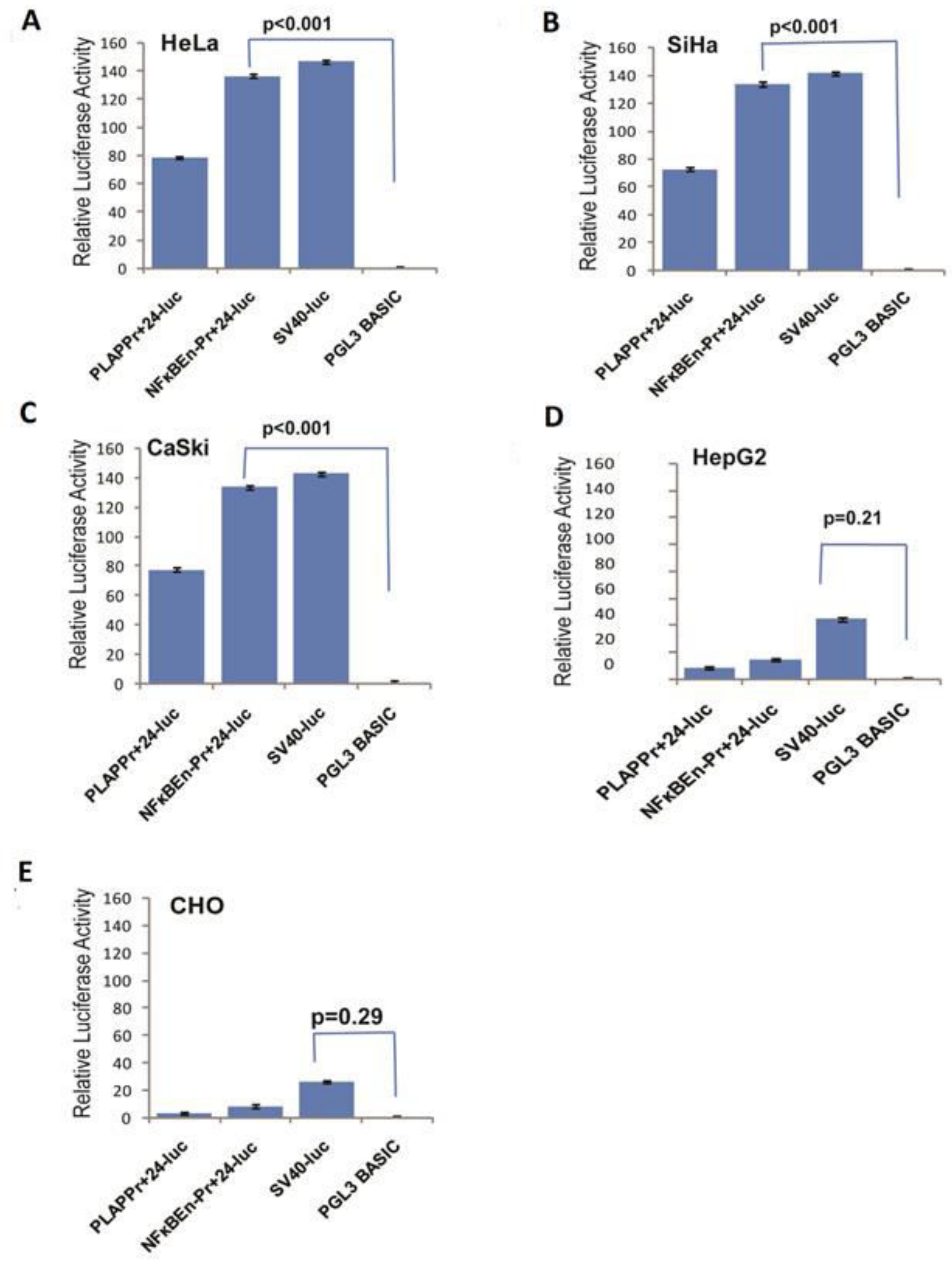

Supplementary Figure S5A: The transcriptional activity and specificity of various luciferase constructs. (A) PLAP positive HPV-18 integrated cervical cancer cell line (HeLa). **(B)** HPV-16 integrated cell line (SiHa). **(C)** HPV-18 and HPV-16 integrated cell line (CaSki). **(D)** PLAP negative

hepatoma cell lines (HepG2). **(E)** Non- PLAP non-human cell line (CHO) were co-transfected by chimeric virosomes (C-scFv-V) separately in triplicates with luciferase expression vectors (PLAPPr+24–luc,SV40–luc and NFκBEn-Pr+24–luc) and Renilla expression vector (pRL-TK). The luciferase activity of each transfection was normalised by the Renilla reading. The luciferase activity is represented by the ratio of specific promoter over the activity of PGL3-Basic. No luciferase activity was observed in PLAP negative cell lines HepG2 and CHO (D and E) even by tissue nonspecific construct SV40-luc demonstrating specific delivery of packaged cargo only to PLAP expressing cells.
